# Structural and proteomic analyses of vitelline membrane proteins of blackbird (*Turdus merula*) and song thrush (*Turdus philomelos*)

**DOI:** 10.1038/s41598-020-76559-4

**Published:** 2020-11-09

**Authors:** Krzysztof Damaziak, Marek Kieliszek, Dariusz Gozdowski

**Affiliations:** 1 Department of Animal Breeding, Faculty of Animal Breeding. Bioengineering and Conservation, Institute of Animal Science, Warsaw University of Life Sciences—SGGW, Ciszewskiego 9 Street, 02-786 Warsaw, Poland; 2 Department of Food Biotechnology and Microbiology, Institute of Food Sciences, Warsaw University of Life Sciences—SGGW, Nowoursynowska 159C Street, 02-776 Warsaw, Poland; 3 Department of Experimental Design and Bioinformatics, Warsaw University of Life Sciences—SGGW, Nowoursynowska 159, 02-786 Warsaw, Poland

**Keywords:** Structural biology, Zoology

## Abstract

In this study, we aimed to perform structural and proteomic analysis of the vitelline membrane (VM) of two species birds belonging to the family Turdidae: blackbird (*Turdus merula*) and song thrush (*Turdus philomelos*). We performed structural analyses using scanning electron microscopy. The VM proteins were identified and compared to the best-known chicken VM proteins. According to our results, VM of both species has a typical three-layered structure: the outer layer, inner layer, and the continuous membrane between them. An unusual observation was the finding of “convexity” formed by the inner layer in blackbird. The role of these convex structures is not known, but they can be typical for the species and can be used in their identification. In addition, we identified two proteins in the VM of both species of birds, of which U3KEZ1 FICAL was not previously identified in any other bird species, and the U3JXV8 FICAL protein was confirmed only once in cockatiel parrot VM. The function of these proteins is not exactly known, but their structure shows similarities to the SERPIN proteins that are involved in microbiological defense, i.e., they are immune proteins. This study contributes to the current knowledge about the structure and composition of proteins of VM, especially because similar analyses have never been performed for Turdidae family. Knowledge of the structure and specific proteins of blackbird and song thrush VM can be beneficial in research on ecology and bird biology and also helpful in developing noninvasive and nongenetic identification methods.

## Introduction

Traditional methods of identification of birds primarily focus on the analysis of DNA isolated from blood or other tissues. Such procedures are risky due to the need to catch birds or remove chicks from their nests. Alternatively, DNA samples can be obtained from contour feathers or surfaces of the eggshell^1^. However, the collection of biological material directly from the environment may give questionable results due to cross-contamination^2^. Li et al.^3^ have shown that eggs and birds can be identified with a high probability by using DNA extracted from the vitelline membrane (VM) of the egg yolk. This method in turn is burdened with the possibility that both the DNA of offspring and the DNA of males may appear in the egg^4,5^. Pure maternal DNA can be isolated from granular cells that join the oocyte during ovulation^6,7^. Granular cells can be identified by the morphological analysis and immune staining of the FSH receptor (FSHR)^8^. The relatively small amount of material (granular cells) is a limitation. It is possible to extract DNA of up to 530 ng/VM from a chicken egg weighing about 55 g, whereas the same from a quail egg (of about 10 g), can yield 360 ng DNA/VM^3,9^. In addition, granular cells lose their vitality quite quickly due to apoptosis and general degradation^3^. Therefore, identification of birds using the DNA contained in the granular cells can be conducted for species with relatively high egg weight, and under such conditions where the time from laying to analysis is as short as possible.

Liu et al.^10^ hypothesized that birds can be identified based on the proteomic analysis of the egg content. They demonstrated that the cognition of species-specific proteins that are likely to be associated with the adaptation of birds to different environmental stimuli maybe a suitable tool to replace DNA analysis. Similar conclusions were drawn by Damaziak et al.^11^ who analyzed the possibility of occurrence of species-specific proteins in the VM depending on their breeding and hatching specifications. It was also shown that the structure of VM differs between different bird species^11–13^.

So far, proteomic and structural analysis of VM primarily concerned chicken eggs. Literature has demonstrated that VM is formed from two basic layers: the outer and the inner layers (OL and IL, respectively) between which there is a thin granular “continuous membrane” (CM, lamina continua)^14–17^. IL is formed in the ovary prior to ovulation from the collagen membrane of the follicular epithelium. Kido et al.^14^ demonstrated that chicken IL VM mainly consists of four glycoproteins: GI, GII, GIII, and GIV. OL, which is formed after ovulation from the mucinous secretion of infundibulum glands (the first segment of the oviduct), mainly contains ovomucin, lysozyme C, lectin, VM outer (VMO) I and II^12^. Mann^18^ confirmed the presence of 137 other proteins in the whole VM of a chicken egg. However, subsequent investigations have shown the significant differences in the structure and composition of proteins of chicken and duck egg yolks VM^12^, selected ratites^13^, and birds belonging to the precocial and superaltrical species^11^. Therefore, in this study, we hypothesized that structural and proteomic elements of VM of egg yolk are species-specific as similarly egg shape^19^ and shell pigmentation^20^.

As morphological and proteomic analyses of wild birds’ eggs are very limited, the study has been undertaken to characterize the structure and protein composition of egg yolk VM of two species belonging to the family Turdidae: blackbird (*Turdus merula*) and song thrush (*Turdus philomelos*). To the best of our study, this is the first study to describe and compare the structure and protein composition of VM of nondomesticated birds. We hope that the results obtained may be useful in the future for taxonomic, ecological, and evolutionary research, and will broaden the current knowledge on the morphological and chemical variability of the eggs of the *Aves* cluster.

## Results

### Characteristics of selected parameters of blackbird and song thrush eggs

Table 1 presents the results of the general characteristics of blackbird and song thrush eggs. Blackbird eggs had more weight than that of song thrush eggs but both show similarity in the proportion of yolk weight. The weight of VM in yolk mass was higher, and individual layers (OL and IL) were thicker in the eggs of song thrush than that of a blackbird.

**Table 1:** Results (mean ± SD) of the comparative analysis of the egg and yolk weights and VM characteristics of eggs from a blackbird *(Turdus merula*) and song thrush (*Turdus philomelos*).

Items		Blackbird (*T. merula*)	Song Thrush (*T. philomelos*)	*P*-value
Egg weight	(g)	7.01 ± 0.17	6.06 ± 0.16	< 0.001
Yolk weight	(g)	1.46 ± 0.07	1.28 ± 0.06	< 0.001
Yolk ratio^a^	(%)	20.73 ± 0.52	21.19 ± 0.78	0.136
VM weight	(g)	0.025 ± 0.002	0.030 ± 0.002	< 0.001
VM ratio^b^	(%)	1.73 ± 0.10	2.37 ± 0.14	< 0.001
**VM thickness**				
IL	(μm)	1.74 ± 0.11	2.25 ± 0.12	< 0.001
OL		2.10 ± 0.16	2.56 ± 0.10	< 0.001
Total		5.15 ± 0.28	6.03 ± 0.15	< 0.001

*IL* inner layer, *OL* outer layer, *VM* vitelline membrane, *SD* standard deviation.

^a^Yolk weight ratio to egg weight.

^b^VM weight ratio to yolk weight.

### General architecture of blackbird and song thrush VM

#### Scanning electron microscopy

**Figure 1:**
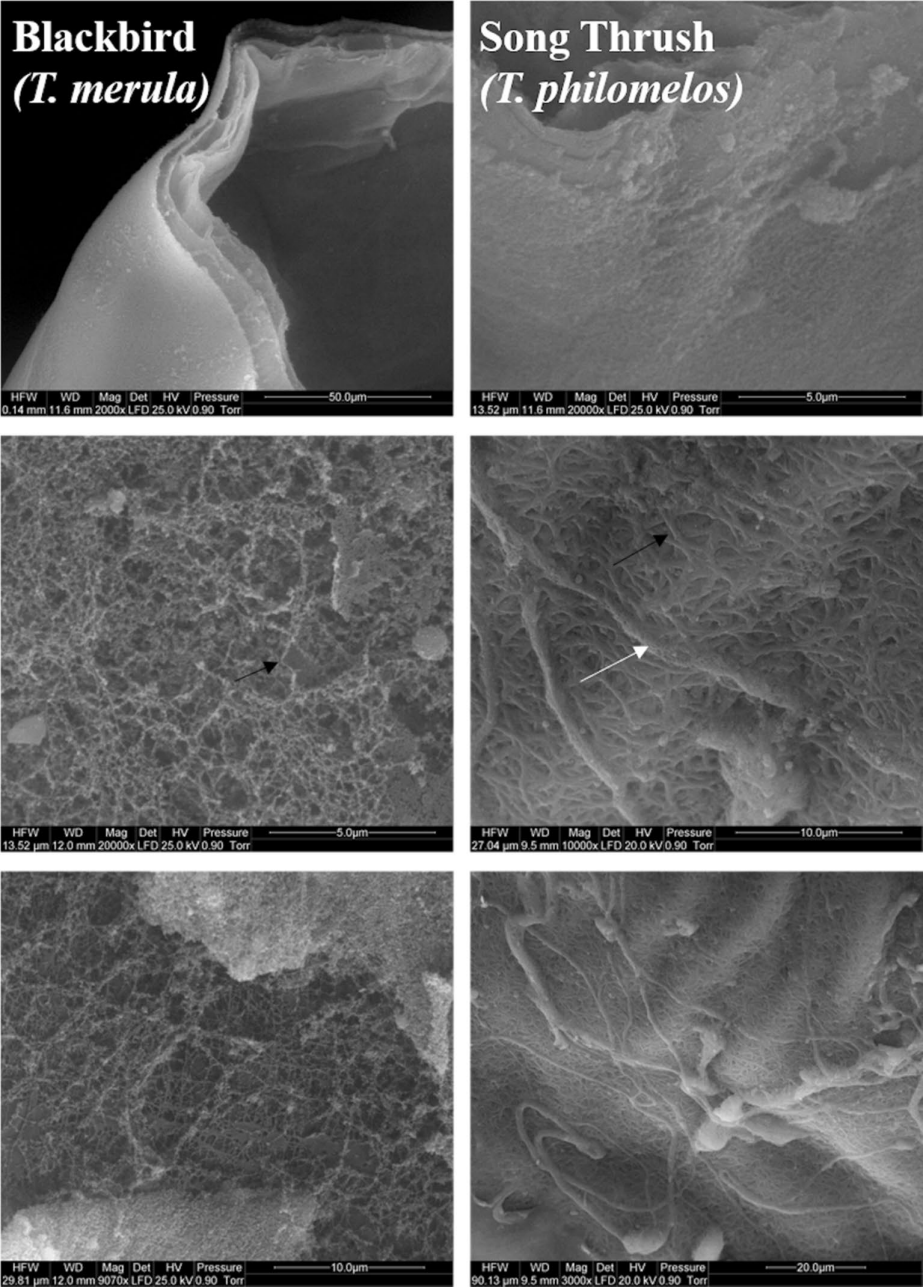
Scanning electron micrograph of outer layers of the vitelline membrane of the egg yolk of blackbird (*Turdus merula*) and song thrush (*Turdus philomelos*). Black arrow shows thin fibers and white arrow shows thick fibers.

**Figure 2:**
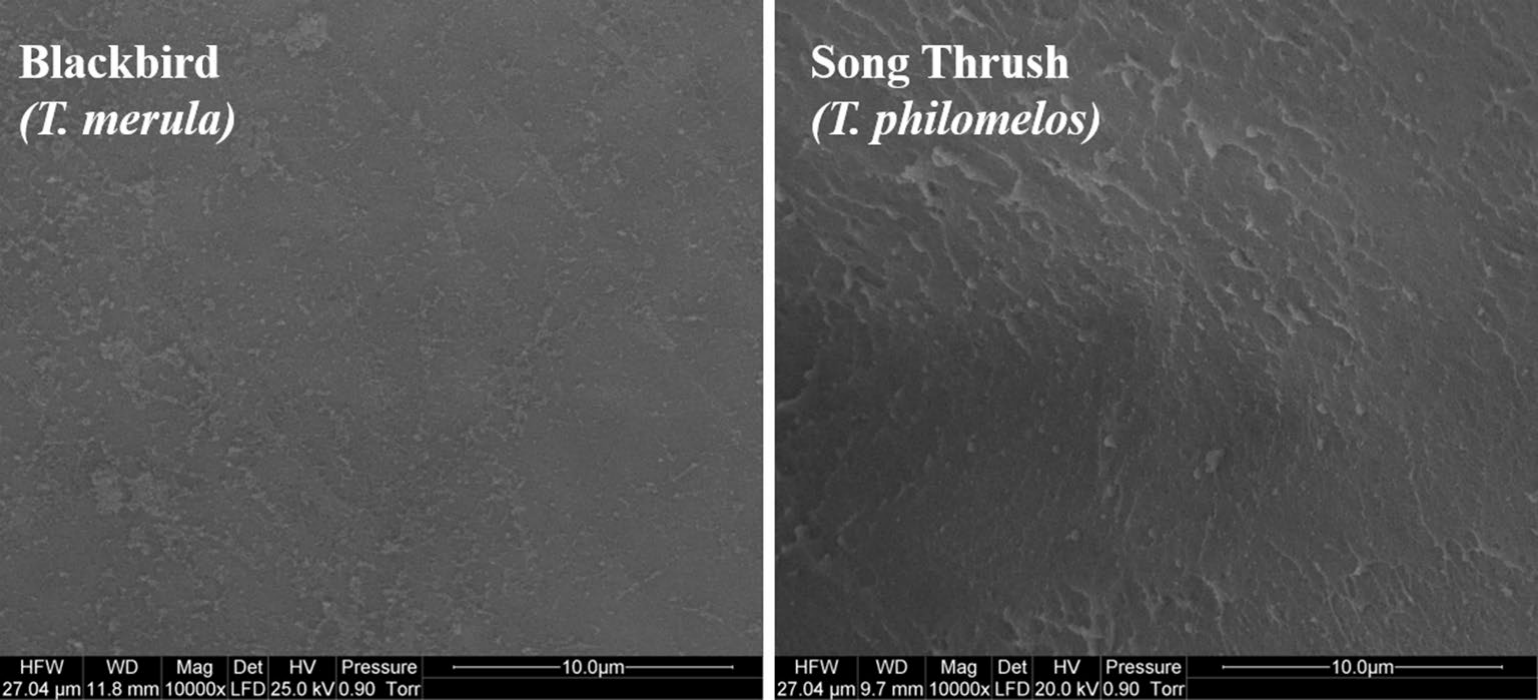
Scanning electron micrograph of inner layers of the vitelline membrane of the egg yolk of blackbird (*Turdus merula*) and song thrush (*Turdus philomelos*).

In this study, the scanning electron microscopic (SEM) image showed that the structure of OL is different for both song thrush and blackbird. In the case of blackbird, OL is formed from thin fibers of similar thickness, whereas in this case of a song thrush, OL is formed from two types of fibers, which significantly differ in thickness (Fig. 1). There were no differences in the structure of IL. In both species, IL is made up of tightly adherent very thin fibers that are invisible even at 10,000 × magnification (Fig. 2; S1 Data).

### General architecture of blackbird and song thrush VM

#### Transmission electron microscopy

**Figure 3:**
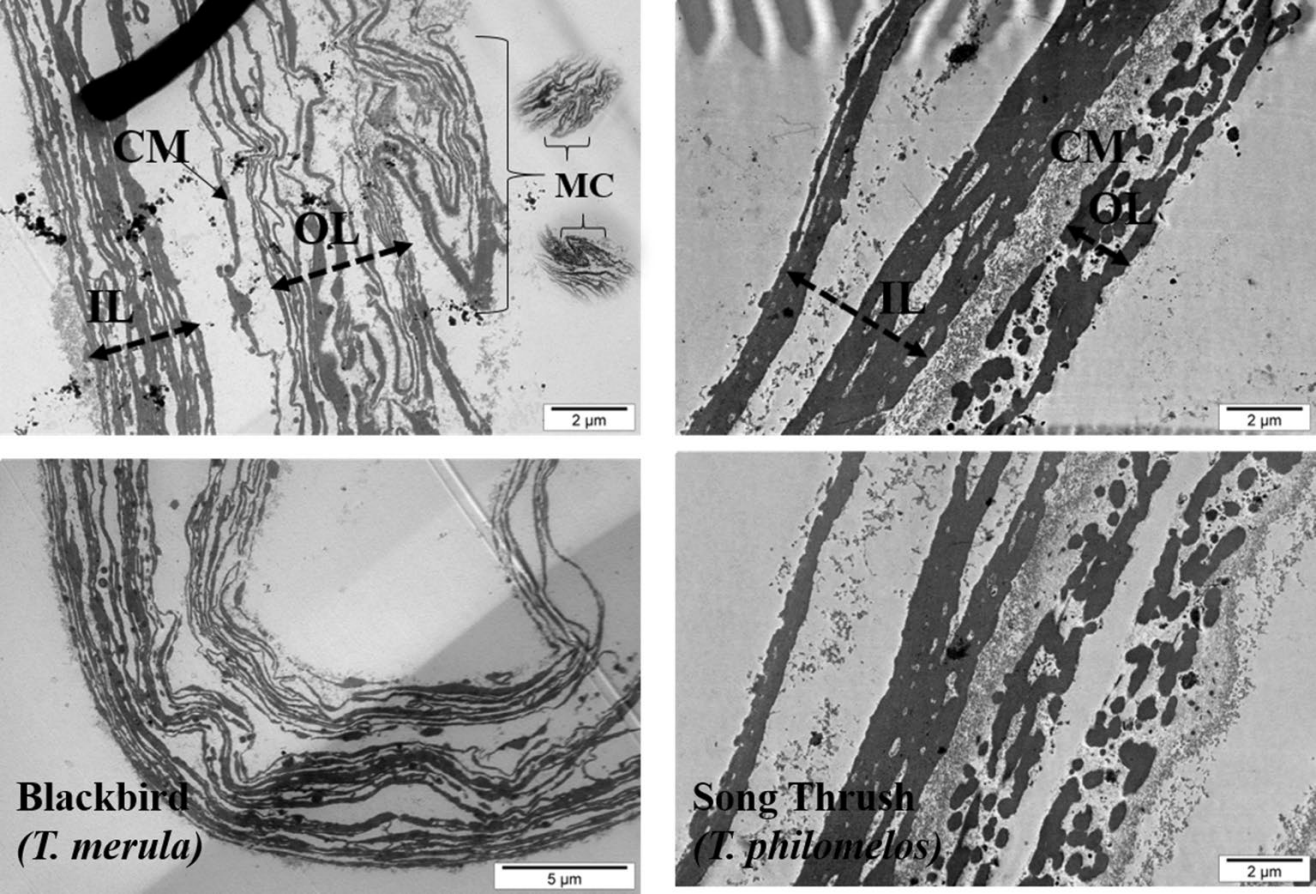
Transmission electron micrographic image of vitelline membrane of the egg yolk of blackbird (*Turdus merula*) and song thrush (*Turdus philomelos*). *OL* outer layer, *CM* continuous membrane, *IL* inner layer, *MC* membrane convexity.

In the transmission electron microscopic (TEM) image, the differences in the VM structure between blackbirds and song thrush are marked. OL and IL in both species are formed from several superimposed sublayers, with single layers thicker in song thrush than that of a blackbird. Moreover, the most external layers of OL in blackbird creates characteristic sharply ended “bumps,” which were not observed in song thrush VM (Fig. 3).

#### Protein of blackbird and song thrush VM

**Figure 4:**
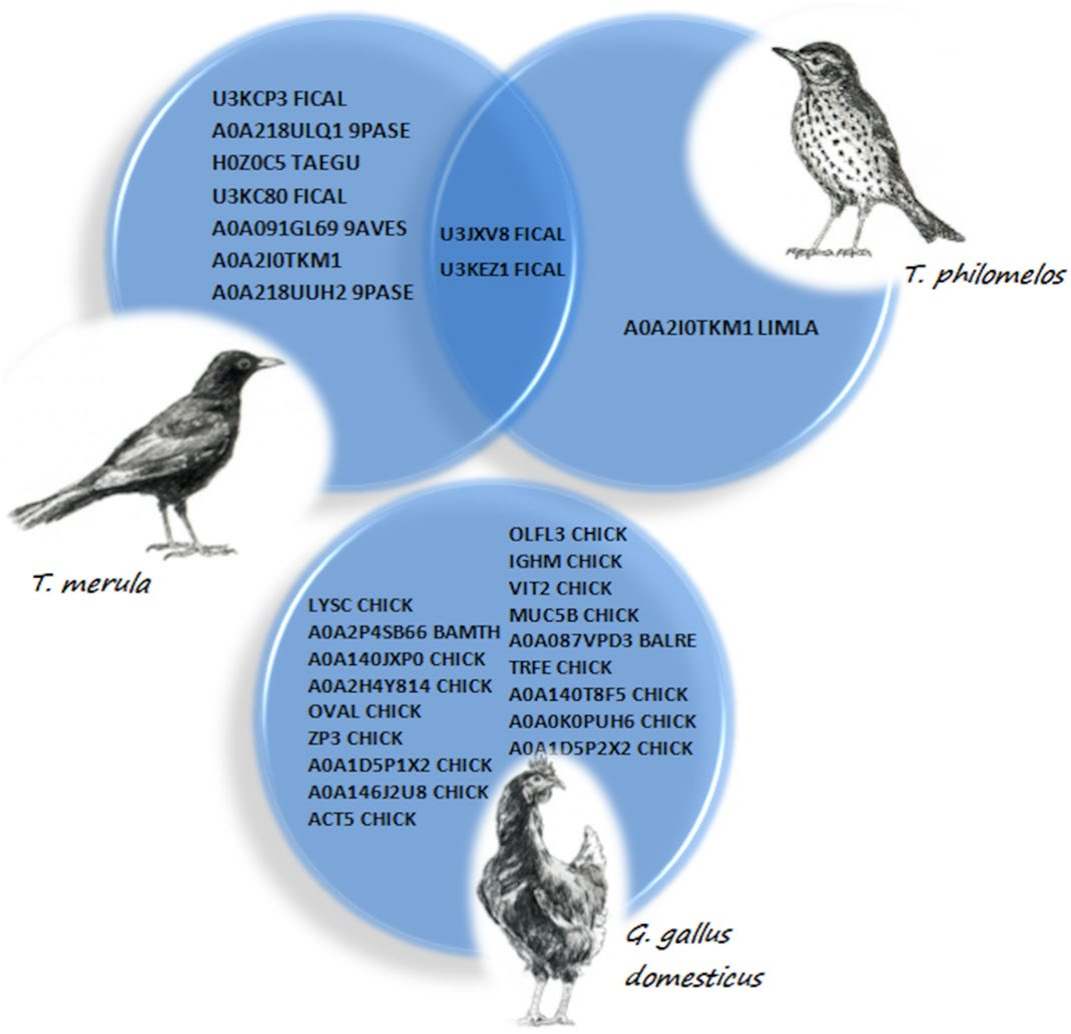
A representative list of protein of the whole vitelline membrane (VM) extracted from the Venn diagrams (a full list is given in Table 2 and S2 Data). The author of the drawings (birds) is Krzysztof Damaziak.

In terms of protein profiles, there were significant differences between individual fractions of blackbird and song thrush VM proteins (Fig. 4). VM obtained from chicken egg (lines 1 and 2) was used as a control. In the case of song bird VM (line 3), about 21 protein fractions were electrophoretically separated. In the case of blackbird VM (line 4), the number of protein fractions was 18. The greatest variability between the VM protein structure of the examined birds was found for fractions with molecular weights ranging from 75 to 100 kDa. The song thrush VM protein fraction (about 50 kDa) had lower molecular weight than that of blackbird VM. The protein fraction with the lowest molecular weight (13 kDa) was found in song thrush VM, although with a very weak intensity, which was absent in blackbird VM.

There were significant differences in the intensity of the bands and the molecular weights of song thrush and blackbird protein fractions in relation to chicken VM protein fractions, which shows that they have a different structural, stratified, or macroscopic structure of VM. In the case of chicken VM, a very high intensity of low-molecular proteins (from 10 to 15 kDa) was found. Moreover, chickens and blackbirds were found to have protein fractions of about 30 kDa, which was not observed for song thrush bird. The high molecular weight protein fractions obtained from chicken VM (100–250 kDa) showed low intensity compared to song thrush and blackbird VM. These differences in protein profiles indicate greater variety among the bird species. Deepening the knowledge about the VM structure of individual birds will help us to better understand its functioning and properties in the future.

**Figure 5:**
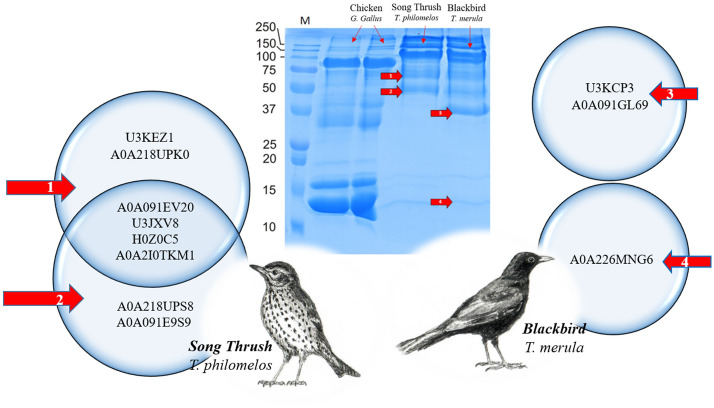
Sodium dodecyl sulfate–polyacrylamide gel electrophoresis of whole vitelline membrane (VM). Analysis of proteins of the VM in the egg yolk of blackbird and song thrush. The red arrows indicate the protein bands selected for detailed analysis. Species-specific list of proteins of the VM extracted from the Venn diagrams (a full list is given in Table 3 and S3 Data). The author of the drawings (birds) is Krzysztof Damaziak.

The 60 kDa protein fraction (arrowheads 1) obtained from the song thrush VM showed the presence of cytoskeletal 5 type II protein and ovalbumin (Tables 2 and 3). The presence of other proteins with previously unknown function and importance in the structure of the VM was also confirmed. The results obtained from the second protein fraction (arrowheads 2) showed the presence of ovalbumins, type II cytoskeletal 5 proteins, in addition to transiently expressed in neural precursor proteins (TENP) and a mixture of uncharacterized proteins (Fig. 5). It is noteworthy that these two fractions had different electrophoretic mobility despite their similarity in terms of protein composition. However, in the case of blackbird VM, similar fractions were confirmed with less intensity.

**Table 2:** Proteomic analysis of water-washed vitelline membranes (VMs) of blackbird (*Turdus merula*), song thrush (*Turdus philomelos*), and chicken (*Gallus gallus domesticus*).

Swiss-prot/trembl accession	Protein	mW (Da)	pI (pH)	PLGS score	Peptides	Theoretical peptides	Coverage (%)	Products	Digest peptides	Protein ID
**Balackbird: *Turdus merul***										
tr	U3JXV8 FICAL Uncharacterized protein OS Ficedulaalbicollis OX 59,894 GN LOC101815176 PE 4 SV	160,510	9.5	2805.4	24	108	13.6	347	347	82,838
tr	U3KCP3 FICAL Uncharacterized protein OS Ficedulaalbicollis OX 59,894 GN ZP3 PE 4 SV 1	45,104	7.1	2104.4	9	23	20.5	113	113	83,304
tr	A0A218ULQ1 9PASE Alpha 2 macroglobulin like protein 1 Fragment OS Lonchurastriata dome	158,124	9.0	2041.8	13	95	9.5	181	181	237,642
tr	H0Z0C5 TAEGU Uncharacterized protein OS Taeniopygiaguttata OX 59,729 PE 3 SV 1	55,310	4.9	1184.3	2	37	3.8	24	24	456,330
tr	U3KEZ1 FICAL Uncharacterized protein OS Ficedulaalbicollis OX 59,894 PE 3 SV 1	44,401	7.3	964.3	6	24	14.5	60	60	27,289
tr	U3KC80 FICAL Zona pellucida glycoprotein 1 OS Ficedulaalbicollis OX 59,894 GN ZP1 PE 4 SV 1	98,809	7.9	558.0	4	32	5.2	66	66	122,034
tr	A0A091GL69 9AVES Zona pellucida sperm binding protein 3 Fragment OS Cuculuscanorus OX 55	32,750	5.4	340.0	1	19	3.3	12	12	585,837
tr	A0A2I0TKM1 LIMLA Type ii cytoskeletal 5 like OS Limosalapponicabaueri OX 1,758,121 GN lla	63,071	5.0	235.4	2	55	2.5	29	29	528,417
tr	A0A218UUH2 9PASE Zona pellucida sperm binding protein 1 OS Lonchurastriatadomestica OX 29	94,707	7.9	163.8	2	31	2.2	29	29	159,428
**Song thrush: *Turdus philomelos***										
tr	U3JXV8 FICAL Uncharacterized protein OS Ficedulaalbicollis OX 59,894 GN LOC101815176 PE 4 SV	160,510	9.4966	1958.895	19	108	11.2578	256	17	82,838
tr	U3KEZ1 FICAL Uncharacterized protein OS Ficedulaalbicollis OX 59,894 PE 3 SV 1	44,401	7.3374	616.8818	5	24	11.9898	48	5	27,289
tr	A0A2I0TKM1 LIMLA Type ii cytoskeletal 5 like OS Limosalapponicabaueri OX 1,758,121 GN lla	63,071	5.0178	205.3799	2	55	2.4735	21	2	528,417
**Chicken;** ***Gallus gallus***										
P00698	LYSC CHICK Lysozyme C OS *Gallus gallus* OX 9031 GN LYZ PE 1 SV 1	16,228	9.2	18,802.8	7	12	36.1	252	7	5495
tr	A0A2P4SB66 BAMTH Uncharacterized protein OS *Bambusicolathoracicus* OX 9083 GN CIB84 01,490	20,279	8.5	12,399.5	14	16	47.0	250	11	789,063
tr	A0A140JXP0 CHICK Zona pellucida sperm-binding protein 1 OS *Gallus gallus* OX 9031 GN ZP1 PE	102,171	8.3	9989.5	12	27	11.0	226	9	290,653
tr	A0A2H4Y814 CHICK OVA Fragment OS *Gallus gallus* OX 9031 GN OVA PE 2 SV 1	42,838	4.9	9123.7	9	27	38.6	185	9	282,735
P01012	OVAL CHICK Ovalbumin OS *Gallus gallus* OX 9031 GN SERPINB14 PE 1 SV 2	42,853	5.0	8592.4	9	27	38.6	186	9	3671
P79762	ZP3 CHICK Zona pellucida sperm-binding protein 3 OS *Gallus gallus* OX 9031 GN ZP3 PE 1 SV 4	46,736	5.9	7070.4	18	22	23.1	225	12	9092
tr	A0A1D5P1X2 CHICK Clusterin OS *Gallus gallus* OX 9031 GN CLU PE 3 SV 1	53,785	5.4	3729.2	15	38	30.5	139	14	319,290
tr	A0A146J2U8 CHICK Protein TENP OS *Gallus gallus* OX 9031 GN TENP PE 2 SV 1	47,387	5.6	2215.3	4	24	11.2	60	4	400,552
P53478	ACT5 CHICK Actin cytoplasmic type 5 OS *Gallus gallus* OX 9031 PE 3 SV 1	41,808	5.1	1481.4	4	34	15.4	31	4	7156
tr	A0A0K0PUH6 CHICK Chemerin OS *Gallus gallus* OX 9031 GN RARRES2 PE 2 SV 1	18,219	9.0	832.9	5	17	27. 8	35	5	344,118
Q25C36	OLFL3 CHICK Olfactomedin like protein 3 OS *Gallus gallus* OX 9031 GN OLFML3 PE 2 SV 1	44,817	5.7	404.5	5	31	11.2	34	5	3280
P01875	IGHM CHICK Ig mu chain C region OS *Gallus gallus* OX 9031 PE 2 SV 2	48,142	6.0	290.5	3	26	11.2	15	3	5166
tr	A0A140T8F5 CHICK Polymeric immunoglobulin receptor OS *Gallus gallus* OX 9031 GN PIGR PE 4	70,526	4.8	260.9	3	42	7.9	25	3	145,853
P02845	VIT2 CHICK Vitellogenin 2 OS *Gallus gallus* OX 9031 GN VTG2 PE 1 SV 1	204,677	9.3	174.8	8	131	5.8	42	8	1087
tr	A0A087VPD3 BALRE Vitellogenin 2 Fragment OS *Balearica regulorumgibbericeps* OX 100,784 G	201,826	9.2	138.2	5	123	3.6	30	5	306,649
P02789	TRFE CHICK Ovotransferrin OS *Gallus gallus* OX 9031 PE 1 SV 2	77,726	6.8	133.3	2	74	4.1	13	2	1940
Q98UI9	MUC5B CHICK Mucin 5B OS *Gallus gallus* OX 9031 GN MUC5B PE 1 SV 1	233,393	5.2	56.8	2	139	1.3	22	2	2906
tr	A0A1D5P2X2 CHICK Alpha-2-macroglobulin-like 1 OS *Gallus gallus* OX 9031 PE 4 SV 1	163,357	8.0	36.1	2	99	1.6	16	2	145,034

All proteins in the whole VMs were identified by sodium dodecyl sulfate–polyacrylamide gel electrophoresis (SDS-PAGE).

**Table 3:** The proteomic analysis of water-washed vitelline membrane (VM) of selected bird species.

Selected bands^a^	Swiss-prot/trembl accession	Protein	mW (Da)	pI (pH)	PLGS score	Peptides	Theoretical peptides	Coverage (%)	Products	Digest peptides	Protein ID
**Song thrush:** ***Turdus philomelos***											
1	tr	U3KEZ1 FICAL Uncharacterized protein OS Ficedulaalbicollis OX 59,894 PE 3 SV 1	44,401	7.3	5009.8	14	24	18.9	152	9	27,289
	tr	A0A218UPK0 9PASE Ovalbumin related protein Y OS Lonchurastriatadomestica OX 299,123 GN S	132,827	4.8	3987.1	12	76	6.3	119	9	236,041
	tr	A0A091EV20 CORBR Ovalbumin Fragment OS Corvusbrachyrhynchos OX 85,066 GN N302 06,297 PE	25,499	4.9	2648.2	3	15	14.4	27	3	396,154
	tr	U3JXV8 FICAL Uncharacterized protein OS Ficedulaalbicollis OX 59,894 GN LOC101815176 PE 4 SV	160,510	9.4	929.4	13	108	9.3	136	12	82,838
	tr	H0Z0C5 TAEGU Uncharacterized protein OS Taeniopygiaguttata OX 59,729 PE 3 SV 1	55,310	4.8	698.8	2	37	3.8	24	2	456,330
	tr	A0A2I0TKM1 LIMLA Type ii cytoskeletal 5 like OS Limosalapponicabaueri OX 1,758,121 GN lla	63,071	5.0	261.7	2	55	2.5	27	2	528,417
2	tr	U3JXV8 FICAL Uncharacterized protein OS Ficedulaalbicollis OX 59,894 GN LOC101815176 PE 4 SV	160,510	9.5	1242.6	14	108	7.3	150	11	82,838
	tr	A0A091EV20 CORBR Ovalbumin Fragment OS Corvusbrachyrhynchos OX 85,066 GN N302 06,297 PE	25,499	5.0	1065.1	2	15	10.0	26	2	396,154
	tr	H0Z0C5 TAEGU Uncharacterized protein OS Taeniopygiaguttata OX 59,729 PE 3 SV 1	55,310	4.8	380.4	3	37	5.7	23	3	456,330
	tr	A0A218UPS8 9PASE Ovalbumin OS Lonchurastriatadomestica OX 299,123 GN SERPINB14 PE 3 SV 1	57,068	5.4	281.2	1	32	3.7	17	1	164,203
	tr	A0A2I0TKM1 LIMLA Type ii cytoskeletal 5 like OS Limosalapponicabaueri OX 1,758,121 GN lla	63,071	5.0	265.2	3	55	4.6	29	3	528,417
	tr	A0A091E9S9 CORBR Protein TENP Fragment OS Corvusbrachyrhynchos OX 85,066 GN N302 00,278	47,380	4.5	221.9	1	22	3.4	10	1	392,396
**Blackbird: *Turdus merula***											
3	tr	U3KCP3 FICAL Uncharacterized protein OS Ficedulaalbicollis OX 59,894 GN ZP3 PE 4 SV 1	45,104	7.1	3178.2	10	23	20.5	142	8	83,304
	tr	A0A091GL69 9AVES Zona pellucida sperm binding protein 3 Fragment OS Cuculuscanorus OX 55	32,750	5.3	614.7	1	19	3.3	20	1	585,837
	tr	A0A2I0TKM1 LIMLA Type ii cytoskeletal 5 like OS Limosalapponicabaueri OX 1,758,121 GN lla	63,071	5.0	307.3	1	55	2.1	14	1	528,417
4	tr	A0A226MNG6 CALSU Uncharacterized protein OS Callipeplasquamata OX 9009 GN ASZ78 006,884 P	49,818	4.9	375.9	3	41	10.6	19	2	510,342

Proteins were identified from the selected bands (^a^according to Fig. 5).

In the case of song thrush, the protein fraction (40–50 kDa) (arrowheads 3), which was not found in blackbird VM, confirmed that it is a mixture of uncharacterized proteins (U3KCP3 FICAL and A0A226MNG6 CALSU), type II cytoskeletal 5 proteins. The additional protein was found in zona pellucida sperm binding protein 3, which is a part of the transparent oocyte casing. Electrophoretic analysis of the next protein fraction (arrowheads 4) revealed the presence of type II cytoskeletal 5 proteins and uncharacterized proteins only (A0A226MNG6 CALSU). Statistical analysis of proteins of song thrush and blackbird VM shows the presence of two proteins (U3JXV8 FICAL and U3KEZ1 FICAL) occurring simultaneously in both species (Fig. 5). These proteins were not detected in the case of chicken VM. Some additional proteins were identified in the whole blackbird VM protein mixture subjected to electrophoresis under denaturing conditions, which were as follows: zona pellucida glycoprotein 1, zona pellucida sperm binding protein 3, zona pellucida sperm binding protein 1, type II cytoskeletal 5, alpha 2-macroglobulin, and uncharacterized proteins (S2 Data).

The analysis of the obtained protein separations on the electropherograms allowed to find significant differences in the protein structure of chicken, song thrush and blackbird VM. Supplementary data (S3 Data) shows result of proteomic analysis.

## Discussion

### VM structure

In this study, we observed several specific features of the blackbird and song thrush VM structure. The most important are the differences in the thickness of the two primary layers (IL and OL) and the presence of “convexity” formed from the most external OL sublayers only in blackbirds. Literature is lacking regarding the VM structure of any species belonging to *Turdidae* family or even the whole *Passeriformes* order. Few studies using eggs of domesticated birds that were published over the last few years have shown that the VM structure is species-specific, and the differences may be related to the difference in their reproductive biology. Chung et al.^12^ observed and described species diversity of the VM structure of birds. They demonstrated that the VM of chicken eggs is made exclusively of different thicknesses fibers, whereas in ducks, there are additionally strongly flattened elements called sheets. Further research using SEM and TEM allowed to find specific structures combining layers of IL and OL in the VM of emu^13^ and an untypical VM formed from 9 rather than 3 layers in eggs of ring-necked pheasant and gray partridge^11^. It is unknown what are the functions of these morphological elements of the VM. However, their occurrence in different forms in different species of birds can be used for identification and opens a new important direction of research for bird biology. Out of 18,043 currently known bird species^21^, the analysis of the VM structure was carried out only for several^3,7,10–17^. Damaziak et al.^11^ were the first and only ones to describe the results of the analysis of the VM structure of two altricial species (cockatiel parrot and pigeon), a group of birds with the same hatching specification as blackbird and song thrush. The other studies concerned only birds belonging to the precocial species, i.e. with a different breeding specification, which is strongly related to the morphological characteristics of eggs^22^. However, on the basis of the VM analysis of these several species, it cannot be confirmed unequivocally whether the VM structure depends on such factors as the number of eggs in a series, the length of incubation, and the advancement of the offspring at the time of hatching. Therefore, this study only allows to increase the knowledge of the VM structure of eggs by another two altricial species and to compare it with a better-known group of precocial species.

### VM protein

The study of the VM structure may be helpful in identifying birds, but the analysis of proteins is a much more precise tool, and it may prove to be species-specific. Proteomic analysis of the whole egg VM protein fractions in this study showed differences in protein profiles obtained after electrophoretic separation under denaturing conditions (SDS-PAGE). In this study, the most important result of the chemical analysis of VM is the isolation of U3JXV8 FICAL and U3KEZ1 FICAL proteins. These proteins are present in both blackbird and song thrush VM but were not detected in chicken VM. A previous study by Mann^18^ also did not confirm the presence of these proteins in chicken VM. Damaziak et al.^11^ isolated U3JXV8 FICAL protein from the VM of cockatiel parrot, but it was not found in the pigeon VM. In this study, the presence of U3KEZ1 FICAL protein was confirmed for the first time as a component of VM of birds. Unfortunately, the exact functions of both the aforementioned proteins are not known and require further analysis. They may be antimicrobial in nature, which provides defense against specific pathogens for a given environment and a specific group of birds. U3JXV8 FICAL protein, isolated from the bird *Ficedula albicollis*, is composed of three domains and has an endopeptidase inhibitor activity (UniProt)^24^. Previously, Guyot et al.^23^ confirmed the presence of a large number of proteins in VM that has antimicrobial activity such as lysozyme and ovotransferrin. Data provided by the Universal Protein Resource (UniProt)^24^ indicate that U3KEZ1 FICAL protein contains SERPIN domain in its structure. The function of such proteins is, among other things, to inhibit the serine and cysteine proteases that act as protective agents. The physiological role of proteins containing the SERPIN domain remains largely unexplored. These proteins can directly bind to bacterial pathogens and cause disorders of the microbial membranes. The consequence of these processes is the formation of pores in the bacterial wall-membrane system that results in the leakage of cellular cytosol into the extracellular environment^25^. Such proteins take part in the processes of cell proliferation, development of extra-embryonic structures, and biomineralization of eggshells^26,27^.

Apart from U3JXV8 FICAL and U3KEZ1 FICAL proteins, analysis of the selected protein bands allowed to identify three other types of ovalbumin in song thrush VM, of which two proteins, namely, A0A218UPK0 and A0A218UPS8 were previously confirmed for white-rumped munia and one A0A091EV20 was characteristic for American crow^28^. It is noteworthy that both bird species, similar to song thrush, belong to the altricial species and these ovalbumins have not been previously confirmed for any of the precocial species. In general, ovalbumin is a protein present in various forms in the eggs of all bird species. Ovalbumin shows a sequence of three-dimensional homology to SERPIN but is not a serine protease inhibitor^29^. The functions of ovalbumin are still the subject of numerous studies but are primarily attributed to the role of storage protein^30^.

In the case of protein band 2 of song thrush, two proteins belonging to type II cytoskeletal 5 were also identified: A0A091E9S9 characteristic for white-rumped munia and A0A2I0TKM1 previously known for bar-tailed godwit^28^. These proteins form the main part of the intermediate fibers, which are the original components of the cytoskeleton. They participate in the structure and organization of the cytoskeleton of the whole VM. Their function is to stabilize and protect the yolk from external mechanical damage. These proteins ensure the maintenance of appropriate tension of VM and are also present in other structures of avian eggs e.g. shell membranes and protein membranes^31,32^. Another protein identified in protein bands 2 of song thrush VM is H0Z0C5, which belongs to intermediate filaments (IFs). Of all proteins identified in this study for song thrush VM, IFs are the least known.

The analysis of protein bands 3 in blackbird VM showed the presence of sperm binding proteins: zona pellucida 3 (U3KCP3) encoded by *ZP3* and A0A091GL69 encoded by *N303_04311*. These proteins, also called sperm receptor in the zona pellucida, bind sperm at the beginning of fertilization and are necessary to initiate the acrosome reaction^33^. In general, the zona pellucida proteins consist of three or four glycoproteins (ZP1-4) with different functions during oogenesis, fertilization, and early embryonic stage. It is believed that oligosaccharides of these glycoproteins play a key role in the recognition of sperm^34^. In this study, zona pellucida glycoprotein 1 (U3KC80 FICAL) was also identified in the VM of blackbird eggs. The last identified protein and at the same time the only protein determined in protein bands 4 of blackbird VM was the protein A0A226MXG6 encoded by *ASZ78_002009*, which has not yet been characterized. Therefore, its function is unknown, and the only species found to contain this protein is the scaled quail precocial species. Thus, it is possible to conclude that this protein cannot be specific to altricial species.

To sum up, this is the first study to describe the structure and protein composition of VM of two species of birds belonging to the *Turdidae* family: blackbirds and song thrush. SEM and TEM analyses widened the knowledge about the structure and composition of VM proteins of birds belonging to the altricial species. New structures of OL in the blackbird VM were observed, which do not occur in any other species in which studies were conducted to characterize the structure of the VM. We also identified two new proteins—U3JXV8 FICAL and U3KEZ1 FICAL—whose functions are still unknown. However, they may be responsible for the defense against pathogens specific to the species or environments in which they live.

## Materials and methods

### Ethics statement

All procedures related to the acquisition of blackbird and song thrush eggs from natural sites as well as their temporary detention, transport, and dismantling for the collection of biological material were approved by the Regional Director of Environmental Protection in Warsaw (PL) on 12 April 2018: WPN-I.6401.102.2018.KZ.2.

All methods were performed in accordance with the guidelines and regulations of the Third Local Ethics Committee on Animal Experimentation in Warsaw (SGGW Warsaw). Due to the analysis of the no incubation eggs, this study did not require direct consent of the National Ethical Commissionat the Ministry of Science and Higher Education in Poland (Directive: 2015/266/EC; Public Information Bulletin, 2017)^35^.

### Egg collection

**Figure 6:**
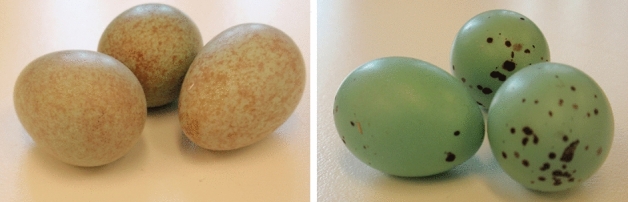
Blackbird (on the left) and song thrush (on the right) eggs before breaking.

Eggs of the blackbird (*T. merula*) and song thrush (*T. philomelos*) species (Fig. 6) were obtained from wild nests between 20 April and 30 May 2018 in central Poland (Mazowieckie Province: 20°43′ E, 52°08′ N). The habitats from which the eggs were obtained are state pine forests or private properties located nearby. Egg collection from private properties took place only with the owners’ consent. A total of 10 eggs were collected for each species, each from a different female (from different nests). Egg collection was carried out in the absence of the female and male near the nest between 14:00 and 18:00. Eggs were collected only from nests containing at least two and no more than three eggs, i.e. before the actual incubation. This was to minimize the risk of nest abandonment and to allow the birds to complete their hatch.

After collection, each egg was wrapped in tethered material and placed in closed PET bags. On-site, no measurements were performed in order to move away from the nest as quickly as possible. After transport to the Institute of Animal Science laboratory (Warsaw, PL), which lasted for about 40 min, the eggs were placed in a cold store at 8 °C for 24 h.

### Measurement of eggs and VM collection

After removal from the cold storage, the weight of each egg was determined (± 0.1 g). Then, the eggs were broken into a separator to separate white from the yolk and the weight of yolk was determined (± 0.1 g). Using a scalpel, VM was cut approximately half the height of the yolk and rinsed in deionized water (~ 4 °C) until the remaining contents were completely rinsed out. The weight of wet VM (± 0.1 g) was determined. Next, the germ disk region was removed from the obtained preparation (not analyzed area). Round fragments (~ 0.5 mm in diameter) were cut out from the remaining VM while avoiding chalaza joints. The obtained samples were secured for further analyses.

### SEM

VM samples intended for scanning micrographs were placed in vials and fixed for 24 h in 6 mL of 3% glutaraldehyde solution + 100 mL of 1% paraformaldehyde in 0.1 M potassium phosphate buffer (pH = 7.2; 4 °C). Re-fixing was conducted in 1% osmium tetraoxide in phosphate buffer at room temperature (18–22 °C) for 1 h. Fixed VM fragments were rinsed with distilled water and dehydrated in a series of ethanol (25, 50, 70, and 95% × 1 each and 100% × 3 each). The samples were dried with CO_2_, mounted on a stub, and coated with gold of 200 Å. The VM structure was observed in SEM FEI QUANTA 200 (Hillsboro, OR, USA), at 25 kV, at various magnifications.

### TEM

The VM samples for transmission micrographs were fixed for 2 h in 2.5% glutaric aldehyde (C_5_H_8_O_2_) and then for 2 h in phosphate-buffered saline (PBS) (pH = 7.2; 4 °C). The final fixation was carried out with 1% OsO_4_ at 4 °C for 1 h. After dehydration in increasing the gradient of ethanol and acetate saturation, the samples were immersed in Epona (812). After polymerization, the VM preparations were cut with a diamond knife on an ultramicrotome (LKB, Sweden) and applied to copper nets, which were then contrasted in uranyl acetate and lead citrate. The VM structure image (TEM) was observed using an electron microscope (JEM 1220 TEM, JEOL, Japan). Selected TEM images were then transferred to a computer and measurements were obtained in Nis Elements, version 5.10. The number of layers forming OL and IL was counted. The thickness of the whole VM, OL, and IL layers was measured and any species-specific structures were marked.

### Protein extraction and gel electrophoresis

Eight eggs from each of the 2 bird species (*T. merula* and *T. philomelos*) and a control sample (*Gallus gallus*) were analyzed, resulting in a total of 16 samples. The proteins were extracted from VM samples using a buffer containing 50 mM Tris–HCl (pH 8.0), 10% glycerol, 2% sodium dodecyl sulfate (SDS), 25 mM EDTA, and protease inhibitor (Sigma-Aldrich, Poland). The samples were shaken on a magnetic stirrer with incubation at room temperature overnight. After the incubation process, the samples were centrifuged (12,000*g*, 30 min, 4 °C) (Eppendorf Centrifuge 5810 R, Germany). The supernatant was obtained and the protein concentration was measured^36^.

The identification of individual protein extracts was performed by electrophoresis under denaturing conditions (SDS-PAGE), which allowed to identify different protein variants^36^. The protein samples were denatured by adding 4 × concentrated Laemmli Sample Buffer (BioRad, Poland) and incubated for 5 min at 95 °C (Eppendorf Thermomixer Comfort, Germany). The samples prepared in this way, together with the protein size marker Page Ruler Plus Prestained Protein Ladder, 10–250 kDa (Thermo Scientific, Poland) were applied to wells of polyacrylamide gel. Electrophoretic separation was performed in Mini-PROTEAN 3 (BioRad, Poland). Briefly, 14% separating gels and 4% thickening gels were prepared. Then, 10 µL of the prepared protein preparations were applied to the wells together with a weighting buffer. Electrophoresis was conducted at a constant voltage of 100 V for about 4 h. The electrophoretic separation was completed when the bromophenol blue dye was about 0.20 cm from the lower edge of the gel. In order to obtain visible bands from proteins, gels were colored with 0.1% Coomassie Brilliant Blue R-250 (Sigma Aldrich, Poland). The gels were discolored in 10% acetic acid and 30% ethanol. Until visualization with Bio-Rad Gel Doc 2000 (BioRad) system, gels were stored in 7% acetic acid.

### Protein identification

After proteins were precipitated, they were dissolved in a 0.1% RapiGest surface-active agent (Waters, Massachusetts, USA) in 50 mM NH_4_OH. After reduction and alkylation of cysteine residues, the proteins were digested using trypsin (Sigma-Aldrich, Poland) at 30 °C for 12 h. The reaction was interrupted by adding trichloroacetic acid (TCA) to the final concentration of 1% (v/v). Low-molecular-weight proteins were additionally digested with chymotrypsin (Sigma-Aldrich, Poland). All digested peptides were analyzed using the nano ACQUITY UPLC system (Waters, Massachusetts, USA) connected to the mass spectrometer. The apparatus was equipped with Symmetry C18 columns (5 μm × 180 μm × 20 mm) (Waters, Massachusetts, USA) to which peptides were added at a flow rate of 10 μL/min in 99% buffer A (0.1% CH_2_O_2_ in water) and 1% buffer B (0.1% CH_2_O_2_ in acetonitrile) for 3 min. The trapped peptides were separated on an analytical column BEH 130 C18 (1.7 μm × 75 μm × 200 mm) balanced in 97% buffer A and 3% buffer B. The column was eluted with a linear gradient of buffer B at a constant flow rate of 300 nL/min at 35 °C. MS-enhanced online analyses were performed in positive ionization mode using the Synapt G2 HDMS mass spectrometer (Waters, Massachusetts, USA). Fragmentation spectra were recorded in the range of 50–2000 Da and the transfer collision energy was increased in the range of 15–35 V. Accuracy of raw molecular weight data was corrected with leucine enkephalin (flow rate 2 ng/μL, 1 μL/min, 556.2771 Da/e [M + H]^+^). Each sample was analyzed thrice and mixed with bovine albumin (60 fmol) as an internal standard during the digestion of tryptic peptides. To identify proteins, peak lists were created from raw data sets and used to search for proteins in the database using the Protein Lynx server, version 2.4 (Waters, Massachusetts, USA). The peptide sequences were identified by an automatic database search using the Mascot search program.

All identified peptides and proteins were tested using ProtParam software^37^.

### Statistical analysis

All the analyzed traits were compared between the species using Duncan’s test at a significance level of *P* ≤ 0.05. Data were analyzed using Statistica 12 software^38^.

## Supplementary information

Supplementary information 1.

Supplementary information 2.

Supplementary information 3.
